# Machine learning approaches for predicting high cost high need patient expenditures in health care

**DOI:** 10.1186/s12938-018-0568-3

**Published:** 2018-11-20

**Authors:** Chengliang Yang, Chris Delcher, Elizabeth Shenkman, Sanjay Ranka

**Affiliations:** 1 Department of Computer & Information Science & Engineering, University of Florida, Gainesville, FL USA; 2 Department of Health Outcomes & Biomedical Informatics, University of Florida, Gainesville, FL USA

**Keywords:** High-cost, High need patients, Machine learning, Predictive modeling

## Abstract

**Background:**

This paper studies the temporal consistency of health care expenditures in a large state Medicaid program. Predictive machine learning models were used to forecast the expenditures, especially for the high-cost, high-need (HCHN) patients.

**Results:**

We systematically tests temporal correlation of patient-level health care expenditures in both the short and long terms. The results suggest that medical expenditures are significantly correlated over multiple periods. Our work demonstrates a prevalent and strong temporal correlation and shows promise for predicting future health care expenditures using machine learning. Temporal correlation is stronger in HCHN patients and their expenditures can be better predicted. Including more past periods is beneficial for better predictive performance.

**Conclusions:**

This study shows that there is significant temporal correlation in health care expenditures. Machine learning models can help to accurately forecast the expenditures. These results could advance the field toward precise preventive care to lower overall health care costs and deliver care more efficiently.

## Background

Health care is one of the largest components of the global economy. According to the World Bank, in 2014, health care expenditures accounted for 9.95% of the world’s total gross domestic product (GDP). Additionally, per capita health expenditures have increased during the last decade. In the United States, the Centers for Medicare & Medicaid Services (CMS) reported that in 2014, health care accounted for 17.5% of the national GDP [1]. This amount is expected to increase over the next several years. because of the expansion of insurance coverage under the Affordable Care Act. In addition, resources, in terms of expenditures, are disproportionately consumed by a relatively small proportion of the health care utilizing population [2]. As a result, this group of health care utilizers has been termed high-cost, high-need (HCHN) patients due to its disproportionate spending concentration [3] and highly prevalent comorbid chronic condition profile [4, 5]. Stakeholders have argued the need for higher efficiency health care, especially for HCHN patients [4]. For example, managed care organizations and health plans (MCOs) have been deployed and funded under capitation payment systems to incentivize health care providers to deliver more cost-effective services [6–8].

Information technology provides a new, promising way to approach a wide-range of health care problems, especially in the “Big Data” era [9]. Health care utilization routinely generates vast amounts of data from sources ranging from electronic medical records, insurance claims, vital signs, and patient-reported outcomes. Moreover, predicting health outcomes using data modeling approaches is an emerging field that can reveal important insights into factors associated with disproportionate spending patterns. Specifically, if we are able to forecast expenditures at the patient-level with good accuracy, we could improve targeted care by anticipating health care needs of HCHN patients. Moreover, predictive modeling can improve our understanding of causal pathways leading to our understanding of expensive events which informs system-level strategies for prevention. Indeed, prevention one of the most effective ways to lower health care expenditures while delivering better quality of care [10–12].

HCHN patients have shown a high degree of persistence in medical expenditures reflected in administrative claims data [2, 13], although some recent studies have concluded that the high utilization may be temporary and not persistent [14]. The purpose of this study is to better understand these temporal patterns and to apply machine learning-based models to predict expenditures. Additionally, we would like to determine if practical timeframes (e.g. 1 month, 6 months) are feasible choices for predicting expenditures.

Approaches to predictive modeling include risk adjustment models that are linear in design and form the basis of many capitation payment systems [6–8]. This models suffer from a number of limitations including use of (1) variables with limited predictive accuracy, (2) specific patient populations or type of care, and (3) population-level models that offer limited information at the patient-level [15–17]. The authors in [18] analyzed the temporal utilization pattern of high utilizers in a large public state insurance program [19]. Studies that attempt to predict HCHN status using patient-level expenditures are lacking. This study aims to address this gap in the literature.

The key contributions of our paper are as follows:

- We used a large longitudinal administrative claims dataset from a public insurance program with millions of enrollees to examine the correlation of patient-level health expenditures across time periods of varying length.
- We applied machine learning models to predict future health expenditures, especially for the HCHN patients, defined here as being in the top 10% of expenditures. The methods scale to input variables of thousands dimensions and millions of patients. The findings indicated that health care expenditures can be effectively predicted (overall R-squared $>\,0.7$). The prediction error for the HCHN patients is lower than the general population, suggesting better model performance for HCHN patients.
- Contributions of input variables to explaining model variance were quantified for each single prediction so that model users can identify potentially modifiable risk factors for possible intervention.

The remaining sections of the paper are organized as follows: In “Methods” section, we introduce the dataset and preprocessing methods used for the study. “Results” section presents our examination of the temporal correlation of expenditures and a detailed explanation of the mechanisms involved with deployed machine-learning models. “Discussion” section provides descriptive results of the temporal correlation of health expenditures and the predictive modeling results. Here, we discuss multiple strategies to better predict expenditures. In “Conclusions” section, we provide our overall conclusions and future directions.

## Methods

### Data and preprocessing

#### Data

*Study population* This study examines administrative insurance claims from the Medicaid program of the state of Texas which has approximately the third largest Medicaid population (annual enrollment of 4.7 million) in the United States [20]. During the study period (2011–2014), there were 1,734,896 adults (ages 18–65) enrolled in the Texas Medicaid program for at least one month. To be included in any analysis, enrolled status needed to be maintained for more than two-thirds of the time for any period-of-interest (either observed or forecasted). Minimum enrollment criteria are applied to avoid including patients enrolled for very short periods of time with highly-variable health care profiles relative to the general medicaid population. For this population, total medical expenditure was defined as the sum of professional, institutional, and dental claims. Pharmacy expenditures were not included in this study. During the study period, the Texas Medicaid program was structured as both fee-for-service (FFS) and MCO-based payment models while FFS was being phased out. For both models, we used the final paid amounts to represent expenditures.

Variables available included diagnosis codes (International Classification of Diseases, Ninth Revision, Clinical Modification, ICD-9-CM), procedure codes (ICD-9-CM procedure codes, Current Procedural Terminology [CPT] and Healthcare Common Procedure Coding System [HCPCS]), and medication codes (National Drug Codes [NDC]). During the study period, 3233 unique ICD-9-CM procedure codes, 21,374 unique ICD-9-CM diagnosis codes, 21,603 unique CPT and HCPCS codes, and 28,366 NDC codes were identified. This study was approved by the IRB of the University of Florida.

*Chronic condition cohorts* We examined the temporal correlation of health expenditures among entire study population as well as four chronic disease cohorts (diabetes, chronic obstructive pulmonary disease [COPD], asthma and hypertension). The difference in correlation strength among chronic disease cohorts as compared to general population was assessed. We identified these clinical cohorts using the Clinical Classifications Software (CCS) [21], using all diagnostic codes, as follows: diabetes (CCS category 49 and 50), COPD (CCS category 127), asthma (CCS category 128) and hypertension (CCS category 98 and 99).

#### Objectives

We constructed predictive models to forecast patient expenditures based on data from prior time periods-of-interest. We examined three prediction objectives (i.e., outcomes):

- Per member per month dollar amount (PMPM, total medical expenditure divided by number of months enrolled in medicaid). This measure is commonly used for expenditure analyses in medicaid programs [22].
- Per member per month dollar amount with log base 10 transformed, *log*PMPM).
- Rank percentiles of the per member per month dollar amount (*pctl*PMPM). This is a continuous measure obtained by dividing the descending ordered rank of PMPM by the number of enrollees in the dataset. Values range from 0 to 1.

Various periods-of-interest were examined in the analysis using periods of 1 month, 3 months, 6 months and 12 months. These time periods are commonly used in studies of health care utilization.

#### Predictors

We designed multiple features as the input variables of the predictive models. For each previous time period consistent with the desired forecast time period:

- Diagnostic codes (ICD-9-CM) grouped into CCS categories (283 categories) [21].
- Procedures codes (CPT and HCPCS) grouped into CCS [21] categories (231 categories).
- Medication information represented by National Drug Codes (NDC). These are grouped by pharmacy classes (893 classes) provided by the U.S. Food and Drug Administration (FDA)’s NDC Directory (Updated Oct. 20, 2015).
- Demographic variables such as age, sex, race/ethnicity (White, Black, Hispanic, American Indian or Alaskan, Asian, Unknown/Other), and disabled status.

After inputting these features, each model consists of approximately 1300 input variables.

### Experiment setup

To examine the temporal correlation of patients total medical expenditures between consecutive time periods (1 month, 3 months, 6 months, 12 months), we used the Pearson product-moment correlation coefficient [23]. We also tested the temporal correlation for four clinical cohorts of chronically-ill patients (diabetes, COPD, asthma and hypertension) prevalent in Medicaid populations of the United States [24]. Then we constructed predictive models to forecast expenditures based on previous expenditures, diagnoses, medical procedures and medications. Details are described as follows:

#### Correlation test

We used a two-step process to test the Pearson product-moment correlation between expenditure rank percentiles using the four aggregations of time periods (1 month, 3 months, 6 months, and 12 months):

Step 1: Rank order all the patients in period 1 and period 2 based on PMPM expenditures.
Step 2: Compute the Pearson product-moment correlation coefficient between the rank percentiles in the two periods.

#### Predictive models

Four predictive models are applied to forecast the patients’ expenditures based on the previous time periods, including ordinary least squares linear regression (LR), regularized regression (LASSO), gradient boosting machine (GBM), and recurrent neural networks (RNN, a deep learning approach). Futoma et al. [25] compared these models in depth for predictive tasks in medicine. The following section describes the details for these models.

*Ordinary least squares linear regression (LR)* Regression is the most widely used method in predictive modeling. It serves as the base risk-adjustment model [6, 7] for modeling risk-based payment systems in health care. Using the input variables as described above, we fit a LR model using least squares to predict future expenditures.

*Regularized regression (LASSO)* Regularized regression, as known as the least absolute shrinkage and selection operator (LASSO) [26], fits a regular linear regression model but penalizes solutions with a large number of nonzero coefficients at the same time. It is broadly utilized as the default approach in many supervised machine learning tasks. Given *M* training instances $\{(\mathbf x _{i},y_{i}),\quad i=1,2,...,M\}$, where $\mathbf x _{i}\in \mathbb {R}^{N}$ is a N-dimensional input variable vector, $y_{i}$ is the predicting objective, $L_{1}$ regularized regression tries to minimize the objective function below:

1 $$\begin{aligned} \min _{\theta }\sum _{i=1}^{M}||y_{i}-\theta \mathbf x _{i}||_{2}^{2}+\beta \Arrowvert \theta \Arrowvert _{1} \end{aligned}$$

where $\theta \in \mathbb {R}^{N}$ are the linear coefficients. The first term of the equation above is the objective function that LR minimizes during optimization. The regularizing term $\Arrowvert \theta \Arrowvert _{1}$ ensures that a large number of entries of $\theta$ are driven to zero. This property is favorable in our case because it makes the model robust to high-dimensional input and selects the most influential input variables. In our study, we use the implementation of LASSO provided by the original authors of the method [26]. A tenfold cross validation approach is used to select the hyper-parameter $\beta$.

*Gradient boosting machine (GBM)* Gradient boosting [27] is another set of successful machine learning techniques that can handle high dimensional input variables. It generates an ensemble of decision trees $f_{t}$ to be used as the predictive model. It learns these trees in an additive manner. In each round, it learns a new tree $f_{t}$ by optimizing the objective function of:

2 $$\begin{aligned} \min _{f_{t}}\sum _{i=1}^{M}\left( g_{i}f_{t}(\mathbf x _{i})+\frac{1}{2}h_{i}f_{t}^{2}(\mathbf x _{i})\right) +\gamma T+\lambda \sum _{j=1}^{T}w_{j}^{2} \end{aligned}$$

where $g_{i}$ and $h_{i}$ are the first- and second-order derivatives of the loss function, which, in our case, the squared error between predicted and true values. *T* is the number of leaves in the decision tree $f_{t}$ and $w_{j}$ are the leaf weights. The last two terms are regularizers to limit mode complexity.

One advantageous property of GBM is that the information gain of the nodes in the decision trees can be aggregated as a measure of input variable importance, which is similar to the coefficients in LASSO. This enables interpretability of tree methods in applications. In practice, we use the implementation of GBM provided by [28]. We trained 1000 decision trees for each GBM. We perform a grid search and fivefold cross validation to optimize the choices of other hyper-parameters such as learning rate and tree depth.

*Recurrent neural networks (RNN)* Recurrent neural networks are a set of deep learning models designed to process sequential data. These models have proven to be very effective in dealing with a variety of sequence tasks, such as speech recognition [29], machine translation [30], sunspot number prediction [31] and video understanding [32]. In health care, RNN models have been used for early detection of heart failure onset from electronic health records [33]. The health claims dataset used in our case to predict medical expenditures could be organized as sequential events (e.g. date of diagnosis, date of procedure, and date of medication use). Thus, we apply RNN to model these events as time series to take advantage of the chronological order, rather than including them in the models as unordered events.

**Fig. 1:**
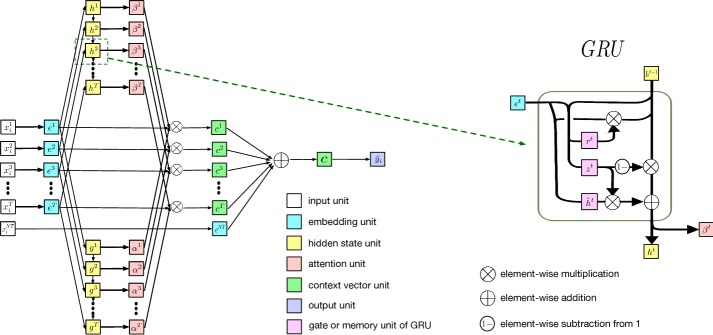
Schematic diagram of the deployed RNN model. The whole process consists of several steps. Step 1: Input variables are embedded; Step 2: A RNN with single gated recurrent unit (GRU) layer is used to generate attention from the sequential embeddings; Step 3: Attentions and embeddings are summed to make the context vector. The context vector is later transformed to output

For a patient $\{(\mathbf x _{i},y_{i}),\quad i=1,2,...,M\}$, where $\mathbf x _{i}\in \mathbb {R}^{N}$ is the input variable vector, $y_{i}$ is the predicting objective, we assumed that $\mathbf x _{i}$ consists of *T* periods. Each period $\mathbf x _{i}^{t}$ is a *K* dimensional vector of input variables. Also, for non-temporal input variables such as demographics, we denote it as a vector $\mathbf x _{i}^{NT}$ of dimension *L*. Thus, $\mathbf x _{i}=\{\mathbf{x }_{i}^{1},\mathbf x _{i}^{2},...,\mathbf x _{i}^{T},\mathbf x _{i}^{NT}\}$. We use a RNN with similar structure of [34] to perform a regression task to predict $y_{i}$. The network structure is described in Fig. 1.

The model takes a three-step approach to make predictions in the following:

- Step 1: To reduce the dimensionality of input, $\{\mathbf{x }_{i}^{1},\mathbf{x }_{i}^{2},...,\mathbf{x }_{i}^{T}\}$ and $\mathbf x _{i}^{NT}$ are mapped to *E* dimensional embedding vectors of $\{e^{1},e^{2},...,e^{T}\}$ and $e^{NT}$ using embedding matrices $W_{T}\in \mathbb {R}^{E \times K}$ and $W_{NT}\in \mathbb {R}^{E \times L}$ respectively, i.e.
  3 $$\begin{aligned} e^{t}= & {} W_{T}{} \mathbf x _{i}^{t} \end{aligned}$$
  4 $$\begin{aligned} e^{NT}= & {} W_{NT}{} \mathbf x _{i}^{Nt} \end{aligned}$$
- Step 2: A RNN with single gated recurrent unit (GRU) layer [35] is used to generate attention weights from the sequential embeddings $\{e^{1},e^{2},...,e^{T}\}$. Attention is a mechanism in deep learning introduced in machine translation [36] and visual recognition [37] tasks that can dynamically decide which part of the sequence should be assigned additional weights. Our model contains two kinds of attention:
  - $\alpha ^{t}$ is scalar that determines that weight of period *t*.
  - $\beta ^{t}$ is an *E* dimensional vector that determines the importance of elements in each embedding $e^{t}$.
  In the GRU layer, recurrent hidden state $g^{t}$ and $h^{t}$ is used to generate $\alpha ^{t}$ and $\beta ^{t}$ respectively. The right panel of Fig. 1 describes the process used to generate $\beta ^{t}$. The same process is applied to generate $\alpha ^{t}$. The intermediate memory unit $\hat{h}^{t}$ takes input from $e^{t}$ and $h^{t-1}$ to update $h^{t}$. The reset gate $r^{t}$ determines which portion of $h^{t-1}$ is absorbed into $\hat{h}^{t}$. The update gate $z^{t}$ determines the weights of $\hat{h}^{t}$ and $h^{t-1}$ when generating $h^{t}$. Formally, the updating rules for $r^{t}$, $\hat{h}^{t}$, $z^{t}$, $h^{t}$ and $\beta ^{t}$ are described as follows.
  5 $$\begin{aligned} r^{t}=\, & {} \sigma (W_{r}e^{t}+U_{r}h^{t-1}+b_{r}) \end{aligned}$$
  6 $$\begin{aligned} \hat{h}^{t}=\, & {} \tanh (W_{h}e^{t}+r^{t}\otimes U_{h}h^{t-1}+b_{h}) \end{aligned}$$
  7 $$\begin{aligned} z^{t}=\, & {} \sigma (W_{z}e^{t}+U_{z}h^{t-1}+b_{z}) \end{aligned}$$
  8 $$\begin{aligned} h^{t}=\, & {} (z^{t}\otimes h^{t-1}) \oplus ((1-z^{t})\otimes \hat{h}^{t}) \end{aligned}$$
  9 $$\begin{aligned} \beta ^{t}= & {} \tanh (W_{\beta }h^{t}+b_{\beta }) \end{aligned}$$
  where $\sigma ()$ is the sigmoid function, $\otimes$ and $\oplus$ are element-wise multiplication and element-wise addition, respectively.
- Step 3: After obtaining the attention values $\alpha ^{t}$ and $\beta ^{t}$, context vector can be generated as follows:
  10 $$\begin{aligned} c^{t}=\alpha ^{t}\beta ^{t}\otimes e^{t} \end{aligned}$$
  The term “context” came from the field of natural language processing, indicating that underlying representation contains information from the preceding and succeeding sequences. The context vectors are aggregated with the embedding vector of non-temporal variables $e^{NT}$ and multiplied by the output coefficients to make predictions:
  11 $$\begin{aligned} c= & {} \sum _{t=1}^{T}c^{t}+e^{NT} \end{aligned}$$
  12 $$\begin{aligned} \hat{y}_{i}= wc+b \end{aligned}$$

In our implementation, we used embedding size of 128. Dropout [38] was applied in embedding and context vectors to control overfitting. The dropout ratio was set to 0.5. To learn all the parameters, adaptive learning rate method ADADELTA [39] is used as the optimization method when performing back-propagation.

#### Interpreting predictions

For predictive models in health care, in addition to high accuracy, interpretability is also crucial [34, 40, 41]. Many machine learning models are often regarded as black-box focusing on pure prediction rather than understanding the degree of variances explained by each component of the model or medical cause-effect pathways. To improve interpretability, we applied additional strategies to quantify the contribution from each single input variable so that model users can interpret and diagnose the predictions. Below are the approaches that we took for each model.

*Ordinary least squares linear regression (LR) and regularized regression (LASSO)* For the two linear models, pulling the contributions of each input variable is straightforward. The product of linear coefficients and variable value are readily converted into the contribution of the corresponding variable.

*Gradient boosting machine (GBM)* As in [42, 43], for each single decision tree learned by the GBM, each test instance is assigned to a leaf following a decision path. The decision path consists of splitting nodes described by input variables. The weights of the leaves are assigned back to the splitting nodes on the decision path and weighed by the gain in each node. As a result, the predictors in the splitting nodes receive a portion of the weights. The contributions are the sums of these portions of weights by input variables across all trees.

*Recurrent neural networks (RNN)* Using methods similar to one proposed in [34], we can show that each prediction $\hat{y}_{i}$ made by RNN can be derived from Eqs. 3 to 12, as follows:

13 $$\begin{aligned} \hat{y}_{i}=\, & {} w\left( \sum _{t=1}^{T}\alpha ^{t}\beta ^{t}\otimes e^{t}+e^{NT}\right) +b\nonumber \\= & {} \sum _{t=1}^{T}\sum _{k=1}^{K}x_{tk}\alpha ^{t}w(\beta ^{t}\otimes W_{T}[:,k])+\sum _{l=1}^{L}x_{l}wW_{NT}[:,l]+b \end{aligned}$$

where $x_{tk}$ is the *k*-th element of temporal input variable vector $\mathbf x _{i}^{t}$ and $x_{l}$ is the *l*-th element of non-temporal input variable vector $\mathbf x _{i}^{NT}$.

From the above equation, the contribution of $x_{tk}$ and $x_{l}$ are $\alpha ^{t}w(\beta ^{t}\otimes W_{T}[:,k])$ and $wW_{NT}[:,l]$ respectively. It is worth noting that regular RNN is a non-interpretable black-box model because of the recurrent hidden states. However, in our model, the recurrences here are used to generate attention weights rather than directly to make predictions. Thus, the model is partially interpretable in terms of input variables.

#### Model selection and validation

For models with hyperparameters (LASSO, GBM and RNN), we selected best fitting models using cross-validation as described above. To validate these models on test dataset, we trained the models to predict period *t* and tested the models to predict period $t+1$. During training, the information of period $t+1$ was never accessed. We reported the R-squared and root mean squared error (RMSE) of rank percentiles as the performance measure. Given the policy interests associated with HCHN patients [2, 3], throughout the study, we used the threshold of top 10% of PMPM expenditure to identify HCHN patients. In order to get robust results, we used at least three different *t* values to have multiple times training and testing. We reported the results using the averaged numbers from these multiple experiments. The only exception is for time periods of 12 months. In this case, we didn’t have sufficient data, giving us only one training set and one testing set.

## Results

We first present the temporal correlation of expenditures for the adult population, HCHN, and patients with chronic conditions. We then present the predictive accuracy for each of the modeling methods. We also describe how these models can be used for potential cause-effect analysis for each patient.

### Temporal correlation

We first present our results for the entire adult population, followed by correlation in expenditures of HCHN and cohorts with specific chronic diseases.

**Fig. 2:**
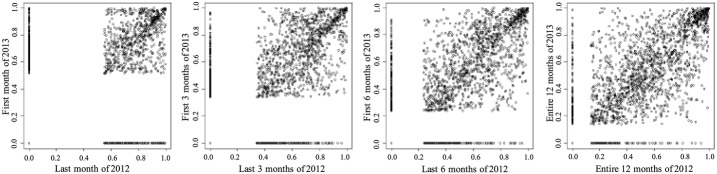
Scatter plot of expenditure percentiles between two consecutive time periods for different period lengths. The upper right corner is denser, implying the HCHN patients are more temporally consistent. From left to right, *x* axis are the last month, last 3 months, last 6 months, and entire 12 months of 2012 respectively. The *y* axis are the first month, first 3 months, first 6 months, and entire 12 months of 2013 respectively

*Entire adult population* The temporal correlation for the adult population is presented in Table 1. A scatter plot of expenditure percentiles between two consecutive time periods for different period aggregations are shown in Fig. 2. The x-axis of each point corresponds to a time period while the y-axis corresponds to an immediate subsequent time period. One can make the following two important observations from Table 1 and Fig. 2. (1) Areas representing HCHN patients are denser, implying HCHN patients have more consistent expenditures than other adults. (2) Correlation is higher with larger period length, which implies patients expenditures are relatively less consistent when the time period is small.

**Table 1:** Average correlation for the entire adult population

Period length (months)	One period later	Two periods later	Three periods later
1	0.564	0.526	0.521
3	0.651	0.584	0.516
6	0.653	0.566	0.515
12	0.676	0.594	–

The correlation increases as the period length increases and decreases as the examined periods become more temporally distant

**Fig. 3:**
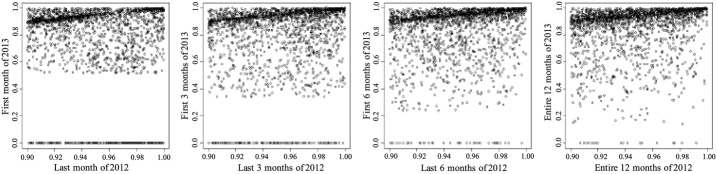
Scatter plot of expenditure percentiles of the top 10% population between two consecutive time periods for different period lengths. The majority of HCHN patients stay above 80% for the next period. From left to right, the *x* axis are the last month, last 3 months, last 6 months, and entire 12 months of 2012 respectively. The *y* axis are the first month, first 3 months, first 6 months, and entire 12 months of 2013 respectively

*Temporal correlation for the top 10% population* Table 2 shows the percentage of the top 10% patients that stayed in the top 10% during the next period. Table 3 shows the average percentile and standard deviation for the top 10% population in the next period. Figure 3 provides a more focused description of the distribution of percentiles of the top 10% in two consecutive periods. This data clearly shows that the expenditures of the top 10% population tend to be consistent. Though a substantial portion of them fell out of the top 10% in the next period, their average expenditure percentiles stayed high. Also, the consistency of high utilization increases for longer durations (at least up to 12 months).

**Table 2:** Percentage of top 10% patients that stayed in top 10%

Period length (months)	One period later	Two periods later	Three periods later
1	45.61	43.10	47.89
3	53.76	50.16	47.57
6	58.38	53.76	51.00
12	61.13	55.25	–

The percentages go up as the period length increases. This suggests that the HCHN patient expenditures are more consistent in longer periods

**Table 3:** Average percentile ± standard deviation in the following periods of the top 10%

Period length (months)	One period later	Two periods later	Three periods later
1	72.19 ± 33.36	68.98 ± 35.88	72.70 ± 33.76
3	80.84 ± 24.42	78.12 ± 27.09	76.58 ± 28.09
6	83.13 ± 21.11	80.60 ± 23.37	79.33 ± 24.12
12	85.39 ± 18.01	82.91 ± 20.19	–

The standard deviation decreases as the period length increases, again suggesting that HCHN patients are more stable in longer periods

**Fig. 4:**
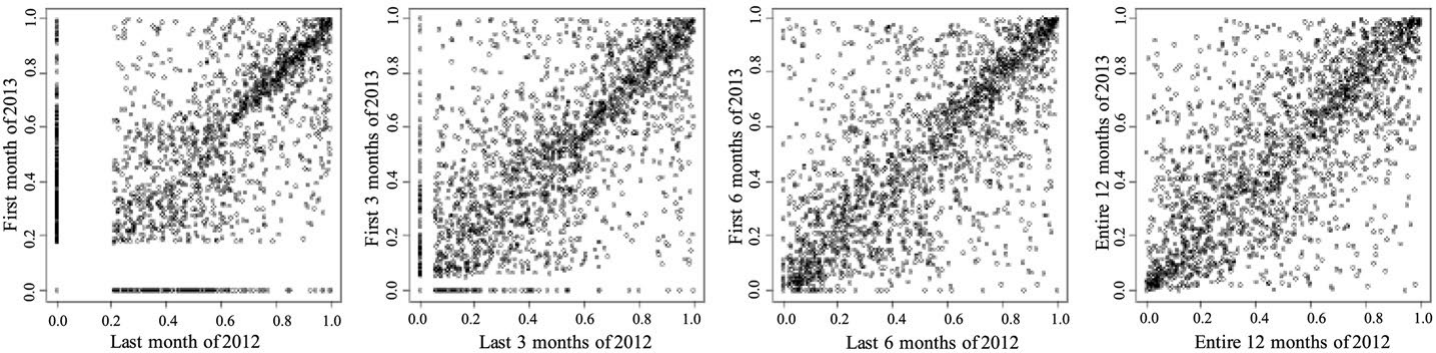
Scatter plot of expenditure percentiles of the diabetes cohort between two consecutive time periods for different period lengths. The conclusions are similar to the entire adult population’s. The HCHN patients in the upper right corner are consistent. The low-cost population in the lower left also shows consistency. From left to right, the *x* axis are the last month, last 3 months, last 6 months, and entire 12 months of 2012 respectively. The *y* axis are the first month, first 3 months, first 6 months, and entire 12 months of 2013 respectively

**Fig. 5:**
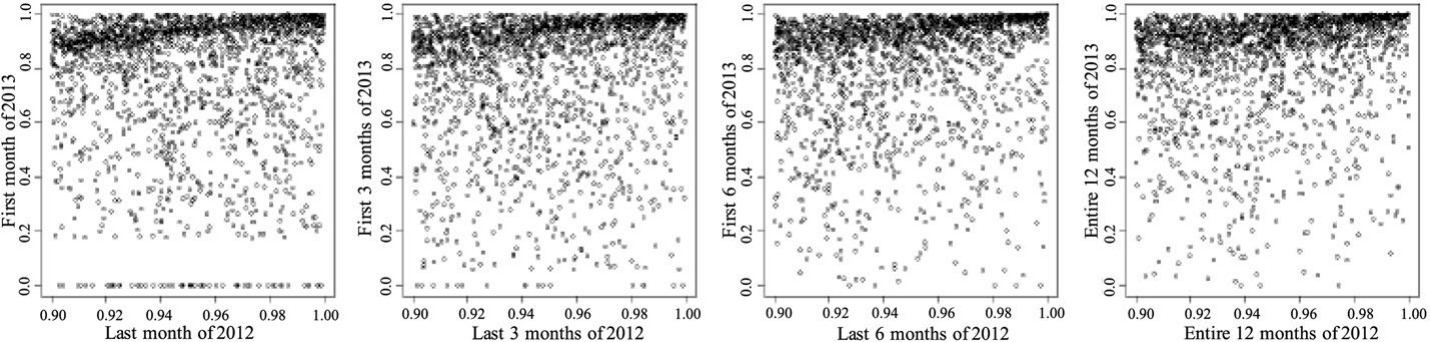
Scatter plot of expenditure percentiles of the top 10% population in the diabetes cohort between two consecutive time periods for different period lengths. HCHN diabetes patients are likely to stay in the top 20%. From left to right, the *x* axis are the last month, last 3 months, last 6 months, and entire 12 months of 2012 respectively. The *y* axis are the first month, first 3 months, first 6 months, and entire 12 months of 2013 respectively

*Chronic conditions cohorts* We investigated four specific chronic conditions prevalent in the Medicaid population of the United States diabetes, COPD, asthma and hypertension. The results showed that a large percentage of the top 10% in the cohort stayed in the top 10% in the next period. We calculated their average percentiles and standard deviation. The counterparts of Tables 1, 2 and 3 and Figs. 2 and 3 for the diabetes cohort are shown in Tables 4, 5 and 6 and Figs. 4 and 5 respectively. The results for patients with COPD, asthma and hypertension are not presented here but the scatter plots for these cohorts are similar to the diabetes cohort.

**Table 4:** Average correlation for the diabetes cohort

Period length (months)	One period later	Two periods later	Three periods later
1	0.611	0.566	0.553
3	0.649	0.592	0.559
6	0.662	0.594	0.541
12	0.675	0.581	-

For one period later, the correlation increases as the period length increases. However, when the subsequent periods are more temporally distant (two or three periods later), this is no longer true

**Table 5:** Percentage of patients from the diabetes cohorts that stayed in the top 10%

Period length (months)	One period later	Two periods later	Three periods later
1	44.28	40.89	39.28
3	45.00	41.31	39.56
6	48.21	44.01	41.63
12	52.66	46.46	–

HCHN patients are more consistent in longer periods (as is the entire adult population)

**Table 6:** Average percentile ± standard deviation in the following periods of the top 10% in the diabetes cohort

Period length (months)	One period later	Two periods later	Three periods later
1	76.33 ± 25.80	74.08 ± 27.43	73.30 ± 27.78
3	78.39 ± 23.24	75.98 ± 24.92	74.93 ± 25.52
6	80.43 ± 21.75	77.95 ± 23.60	76.50 ± 24.50
12	83.07 ± 19.61	79.85 ± 22.27	–

We observe less variation for longer time periods

**Fig. 6:**
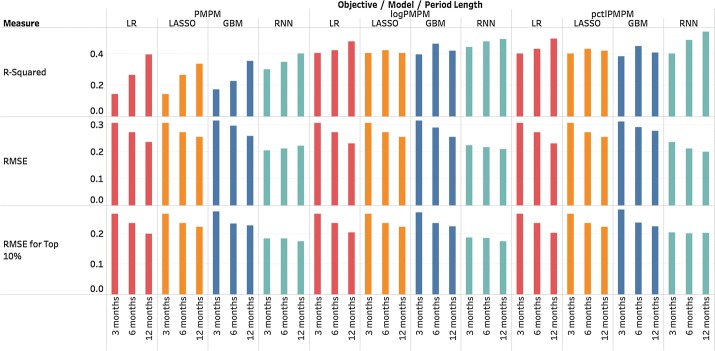
Comparison of different period lengths. Generally performance improves when the time period becomes longer, which is consistent with higher correlation in the same trend. However, GBM and LASSO seem to find their best R-squared when period length = 6 months in predicting *pctl*PMPM and *log*PMPM

Just as we found in the general population, for each disease cohort, we observed a significant correlation in expenditures from one time period to the next. This correlation was stronger for the HCHN population than the general population, and was sustained for longer period lengths.

Table 7 compares the temporal correlation for each of the chronic disease cohorts. We observe that chronic-disease cohorts have similar correlations to the overall adult population. The diabetes cohort has a slightly stronger short-term correlation while the asthma cohort show a slightly stronger longer-term correlation.

**Table 7:** Temporal correlation comparison between cohorts

Period length and lags	General Medicaid population	Diabetes cohort	COPD cohort	Hypertension cohort	Asthma cohort
1 month, One period later	0.564	0.611	0.591	0.588	0.587
1 month, Two periods later	0.526	0.566	0.548	0.543	0.548
1 month, Three periods later	0.521	0.553	0.526	0.530	0.533
3 months, One period later	0.651	0.649	0.611	0.629	0.640
3 months, Two periods later	0.584	0.592	0.554	0.572	0.600
3 months, Three periods later	0.516	0.559	0.526	0.541	0.567
6 months, One period later	0.653	0.662	0.626	0.644	0.680
6 months, Two periods later	0.566	0.594	0.568	0.581	0.624
6 months, Three periods later	0.515	0.541	0.522	0.530	0.585
12 months, One period later	0.676	0.675	0.651	0.664	0.718
12 months, Two periods later	0.594	0.581	0.563	0.570	0.631

The difference in the correlation is small between these cohorts

### Prediction

In this section, results for predicting the expenditures at the patient-level using prior expenditure information is presented. Only expenditure data for the immediate preceding period-of-interest followed by up to 4 subsequent periods and other claims-based information available for the patient is used.

*Baseline* Baseline models below using one prior period-of-interest and only prior expenditures variables (prior PMPM, *pctlPMPM* or *log*PMPM depending on predicting objective) are presented. Table 8 shows the baseline results of quarter-to-quarter prediction. Baseline models fit reasonably-well as indicated by the R-squared values. More than 40% of the variation is explained by the transformed models (*pctlPMPM* and *log*PMPM). RNN is the best model for all measures. The differences between RNN and other models in RMSEs are substantial, implying that expenditures are ranked much closer to the true rankings by RNN.

*Choice of period length* We extended the prediction period, in part, based on preliminary results indicating that Medicaid expenditures are more consistent over longer periods. Figure 6 shows the results for 3 months, 6 months and 12 months. Prediction accuracy results are presented for the test data only. As the period-of-interest increases from 3 to 12 months, fit measures generally improve, with the exception of the R-squared statistic for LASSO and GBM in predicting *log*PMPM and *pctl*PMPM; and RMSE for RNN in predicting PMPM. These results generally show that predictive models are more effective for longer periods. This finding is expected as aggregation over longer periods tends to reduce short-term deviations from the models. Even so, reasonable consistency in expenditures is present from one period to the next, which offers utility in predicting high utilizers in practical scenarios.

**Table 8:** Baseline predictive model results

Predicting objective	Model	Train			Test		
		R-squared	RMSE	RMSE for Top 10%	R-squared	RMSE	RMSE for Top 10%
PMPM	LR	0.145	0.306	0.264	0.141	0.306	0.264
	LASSO	0.145	0.306	0.265	0.141	0.306	0.264
	GBM	0.199	0.317	0.270	0.172	0.314	0.272
	RNN	0.302	0.201	0.180	0.298	0.204	0.183
*log*PMPM	LR	0.401	0.306	0.265	0.402	0.306	0.264
	LASSO	0.401	0.306	0.265	0.402	0.306	0.264
	GBM	0.399	0.316	0.267	0.394	0.314	0.269
	RNN	0.445	0.220	0.184	0.442	0.223	0.187
*pctl*PMPM	LR	0.399	0.306	0.265	0.400	0.306	0.264
	LASSO	0.398	0.306	0.265	0.400	0.306	0.264
	GBM	0.384	0.314	0.275	0.382	0.312	0.277
	RNN	0.405	0.232	0.203	0.400	0.235	0.203

RNN outperforms other models in this case

**Fig. 7:**
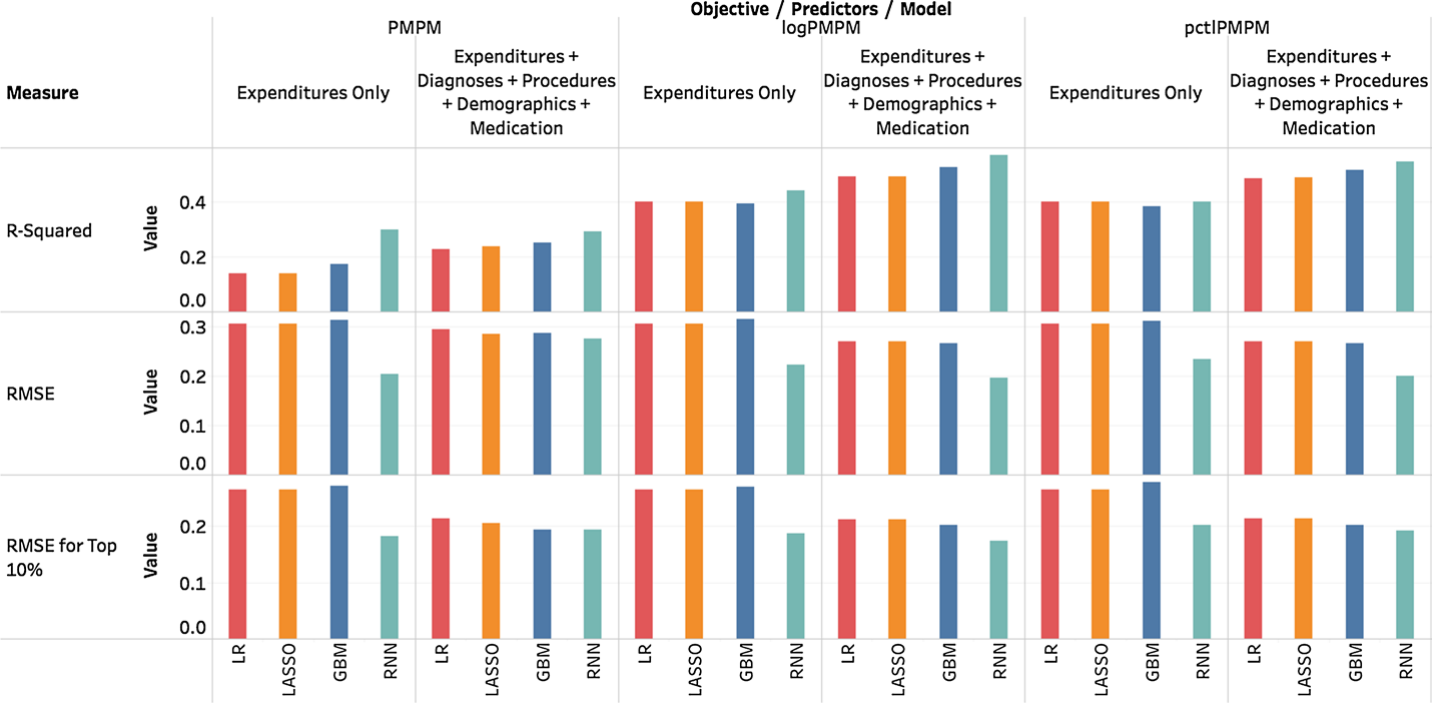
All four models improved after adding demographics, diagnoses, medical procedures and medications as input variables, suggesting that though prior expenditures already provide a good approximation for future spending, additional information is useful in predictive modeling

*Using additional information* In this subsection, we present results after incorporating additional information to our baseline models. In particular, we added Medicaid administrative claims data, such as patient-level demographics, diagnoses, medical procedures and medications. Figure 7 shows performance improvement after adding these inputs during a quarter-to-quarter prediction. Nearly all measures improved substantially with this additional information. When we repeated this procedure for periods of 6 months and 12 months, the improvement persisted. Thus, though historic costs are strong predictors of future costs, additional information, such as demographics, diagnoses, medical procedures and medications, improves prediction accuracy. We note that these models use thousands of variables making overfitting a concern, but the number of individual patients represented in the data approaches 1 million. Thus, as long as the models are well-regularized, the possibility of overfitting is reduced.

**Fig. 8:**
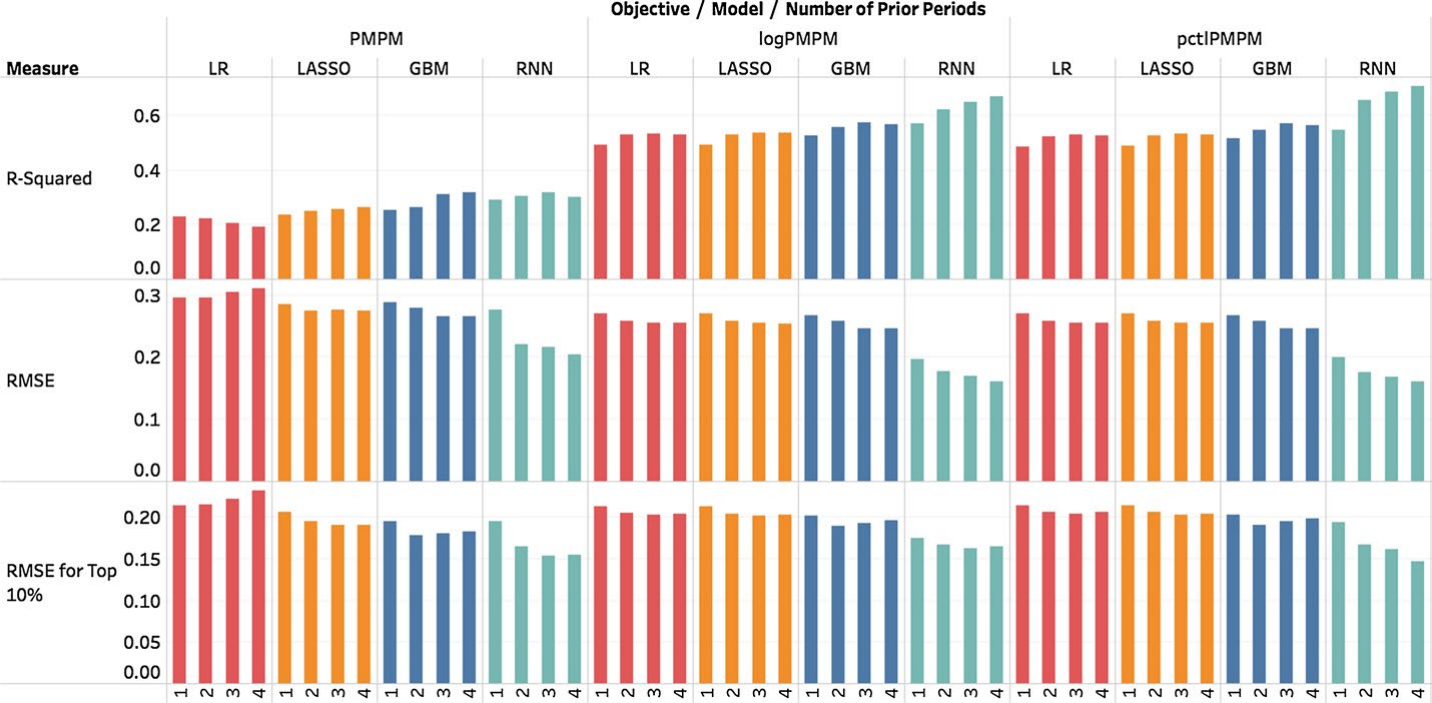
Performance changes after adding more prior periods. Most measures substantially improved after adding the first three periods. The gain for adding a fourth period to LR, LASSO and GBM is minimal. RNN benefits most, indicating its stronger ability to model temporal relations

*Including additional prior periods* In this section, we increase the number of prior periods used for prediction as this should improve prediction performance. Figure 8 shows the performance changes after adding more periods. Only quarter-to-quarter prediction results are presented. Other quarterly inputs include diagnoses, medical procedures and medications. Demographic variables are entered into the models once as they are assumed to be fixed over the study period.

The models appear to reach an improvement ceiling at approximately three prior periods. The RNN model benefits the most by the use of additional periods, which is consistent with literature showing that they are effective in modeling temporal relationships.

The R-squared of the linear models for the testing data decreases when the predicting objective is PMPM. This is likely due to the fact that PMPM is not linearly distributed in the parameter space unlike its transformed versions (*log*PMPM and *pctl*PMPM). Using a large number of parameters and prior periods (effectively inducing a multiplicative effect on the number of parameters) in a linear model increases the likelihood of overfit. We found that the R-squared for the training dataset in the same setting increased with the number of prior periods, which supports our claim of overfitting and preference for models that can adjust this risk, such as LASSO and GBM. The results above were similar with using 6-month periods.

### Interpreting the models

**Fig. 9:**
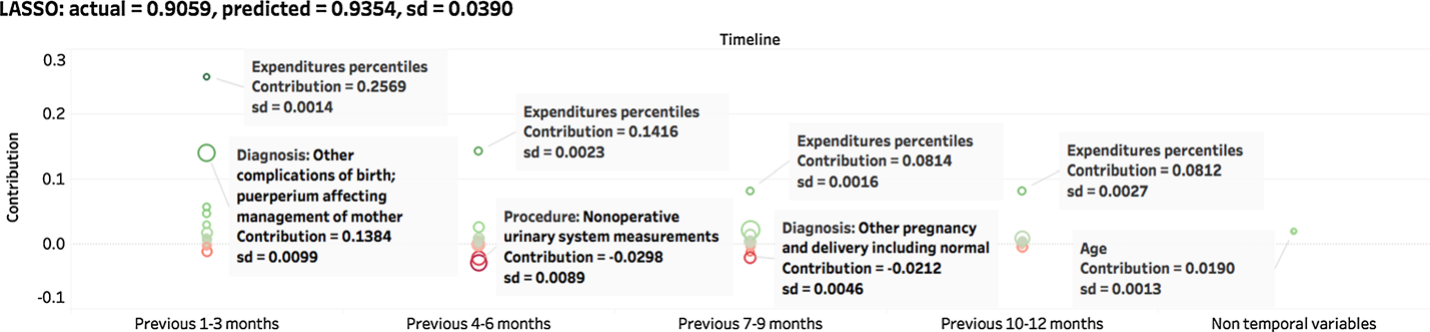
Contributions derived from a prediction by LASSO. The radius of the circle corresponds to the standard deviation of the contribution

**Fig. 10:**
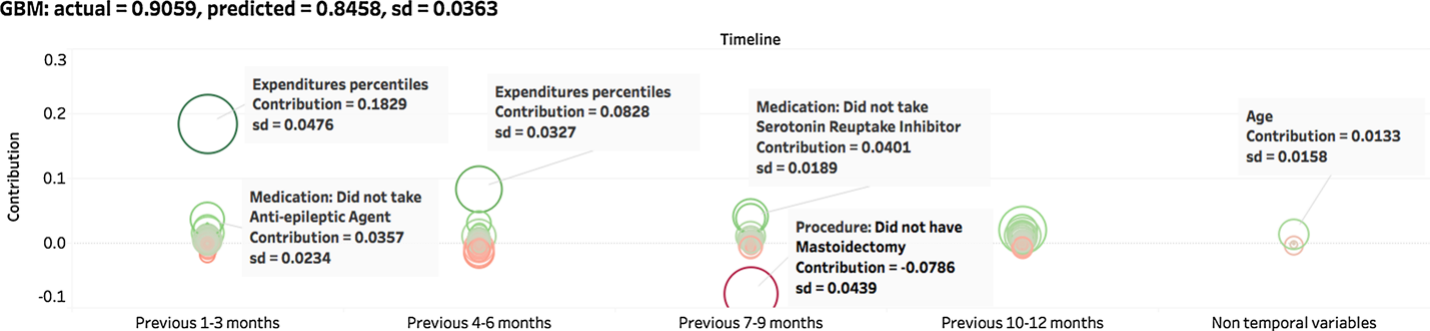
Contributions derived from the same prediction by GBM. We observe a larger variation in contributions. But the variation in predicted value is similar

**Fig. 11:**
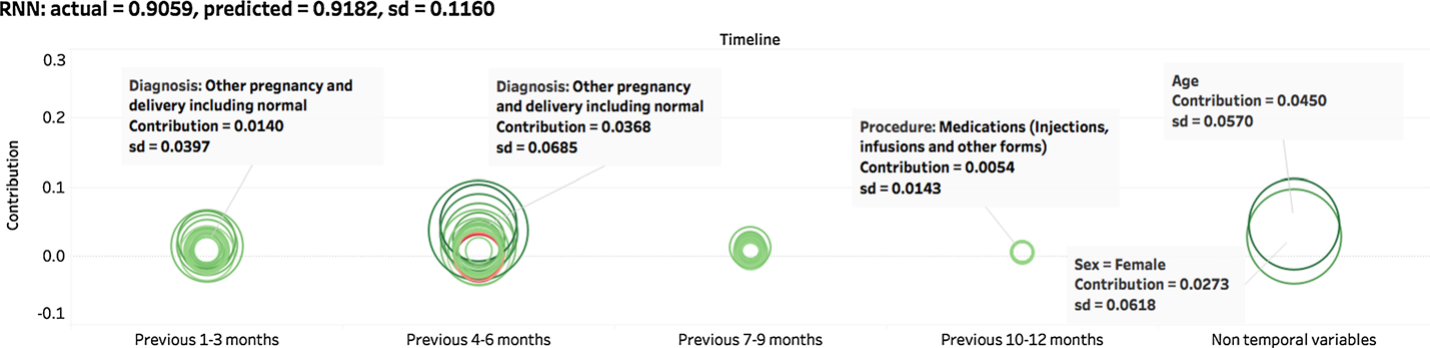
Contributions derived from a prediction by RNN. When comparing LASSO, GBM and RNN, LASSO not only gives stable predicted value, but also generates stable contributions. GBM has consistent predicted values, but is less stable in contributions. RNN is unstable in both, possibly due to its non-convex optimization procedure

For better understanding each prediction, we quantify the contribution from every single input variable in each model used. Figures 9, 10, and 11 present the contributions pulled from LASSO, GBM, and RNN models for the same patient. Both expenditures and additional information for four prior periods were used to train the models. We selected per member per month expenditure percentiles (*pctl*PMPM) as the prediction objective. To examine the variations for the same model, we resampled the training data with replacement for 10 times and trained a different model for each round. These models were then used to make 10 different predictions for the same patient. The average predicted score and its standard deviations (sd) are shown are the graphics. For visualizing contributions, we plotted each input variable as a circle on the timeline. The center and radius of each circle represent the average contribution of the variable and its standard deviation respectively. Results for LR (not presented) are similar to LASSO.

We repeated the process described above for 50 patients and the result for one patient are shown in Figs. 9, 10, and 11. For all these test cases, including the one shown here, the results demonstrates that all three models make robust predictions. LASSO and GBM have comparatively lower standard deviations. LASSO is the most stable model that consistently generates similar contributions. GBM has a larger standard deviation in contributions but can still derive influential ones from all variables. However, the contributions of each variable generated by RNN are very unstable. Clearly this method is not effective for deriving the importance of input variables. Considering that deciding the parameters of RNN requires a non-convex optimization procedure using the stochastic gradient descent may end up with any local minima, it is not surprising that a stochastic algorithm would give different solutions (generally corresponding to a different local minimum) each time, leading to much larger variations in contribution estimates of input variables.

In conclusion, LASSO and GBM are more effective in generating interpretable contributions and find important input variables than the RNN model.

### Choosing the best model

The choice of the best model depends on whether the goal is to best predict expenditure or better understand the contributions of underlying factors. From Figs. 7 and 8, we can conclude that RNN is the best model for prediction. GBM is slightly better in R-squared and RMSE for top 10% than LASSO, but GBM performs similarly with LSAAO for RMSE. However, for clearer interpretation of a particular prediction, LASSO and GBM are more suitable.

In terms of comparing different prediction objectives, RNN seems to perform best using *pctl*PMPM. The reason for this could be that *pctl*PMPM is strictly contained in [0,1], which is less likely to cause significant gradient vanishing or exploding issues that are common in back-propagation when optimizing neural networks. For LR, GBM and LASSO, the choice of predicting objectives is a task-specific decision. If minimal RMSE for top 10% is the goal, one should use PMPM as the predicting objective. If optimizing R-squared is more important, one should consider using *log*PMPM or *pctl*PMPM. All three objectives are similar in overall RMSE.

## Discussion

In 2014, the top 1 percent of expenditures in health care accounted for approximately one-fifth of total health care expenditures [2, 44]. As a result, this group has been termed HCHN patients. Disproportionate spending concentration in this group is also prevalent in other countries [5]. Prior literature [14] has suggested that these expenditures may be episodic and not temporally consistent. If this is indeed the case, the benefit of modeling patterns of high expenditures may be severely limited by a high degree of randomness in their health care utilization.

However, our study clearly shows that health care expenditures are significantly auto-correlated within the Texas Medicaid program. With around 5 million enrollees. Texas has the third largest Medicaid population in the United States. This result may motivate preventive interventions. Auto-correlation suggests an underlying process structure that may be driven by modifiable factors. Thus, highly predictive machine-learning models can enable providers to direct these interventions to the right HCHN population.

This study has several limitations. First, we conducted the study within one states Medicaid program. The results may vary by state and/or payer type. Second, we applied only general-purpose machine-learning models. Some tailored models may have better performance. Third, the predictive models provide little guidance on the preventive factors needed to inform interventions. Finally, health status determined from claims data only is limited. It may be necessary to include additional data sources, such as narrative components of electronic health records (EHR), disease severity measures, and/or social determinants of health.

Future work will address some of these limitations. We plan to expand the analysis to different types of health care programs. We will also collect additional data, mentioned above, to evaluate predictive performance. Moreover, we will collaborate with clinicians and policy experts to make the models clinically-relevant by integrating domain expertise to better direct preventive interventions.

## Conclusions

In this work, we tested the temporal correlation of health care expenditures for multiple time periods. Our results show that health care expenditures are temporally consistent. Further, this correlation is significantly higher for the HCHN patients as compared to the general population. For patients with chronic conditions, the temporal consistency of expenditures was high, but not appreciably higher than the general population. This finding was somewhat surprising, as one would have expected chronic conditions to lead to more consistent expenditures.

Overall, machine learning models are very predictive to forecast health care expenditures. We iteratively developed several predictive models to forecast expenditures. First, we started from a baseline case using only expenditures and step-wisely added input variables to the models. We showed that additional information such as clinical information and demographics are useful to improve prediction performance. In addition, we showed that it is beneficial to have historical data from more prior time periods. The improvements due to additional prior periods saturates after three to four periods. The prediction accuracy of RNN outperforms LR, LASSO and GBM. In terms of prediction interpretability, LASSO and GBM consistently select similar variables and generate stable contributions independent of the resampling process.
